# Features of Postnatal Hippocampal Development in a Rat Model of Sporadic Alzheimer’s Disease

**DOI:** 10.3389/fnins.2020.00533

**Published:** 2020-06-05

**Authors:** Ekaterina A. Rudnitskaya, Tatiana A. Kozlova, Alena O. Burnyasheva, Anna E. Tarasova, Tatiana M. Pankova, Marina V. Starostina, Natalia A. Stefanova, Nataliya G. Kolosova

**Affiliations:** ^1^Laboratory of Molecular Mechanisms of Aging, The Federal Research Center Institute of Cytology and Genetics, Siberian Branch of the Russian Academy of Sciences (SB RAS), Novosibirsk, Russia; ^2^Laboratory of Central Mechanisms of Regulation, Institute of Molecular Biology and Biophysics, Federal Research Center of Fundamental and Translational Medicine, Novosibirsk, Russia

**Keywords:** Alzheimer’s disease, postnatal development, aging, hippocampus, neurogenesis, hippocampal mossy fibers, OXYS rats

## Abstract

Aging is the major risk factor of the most common (∼95% of cases) sporadic Alzheimer’s disease (AD). Accumulating data indicate middle age as a critical period for the relevant pathological processes, however, the question of when AD starts to develop remains open. It has been reported only recently that in the early postnatal period—when brain development is completing—preconditions for a decrease in cognitive abilities and for accelerated aging can form. Here, we hypothesized that specific features of early postnatal brain development may be considered some of the prerequisites of AD development at an advanced age. To test this hypothesis, we used OXYS rats, which are a suitable model of sporadic AD. The duration of gestation, litter size, and weight at birth were lower in OXYS rats compared to control Wistar rats. The shortened duration of gestation may result in developmental retardation. Indeed, we noted decreased locomotor activity and increased anxiety in OXYS rats already at a young age: possible signs of altered brain development. We demonstrated retardation of the peak of postnatal neurogenesis in the hippocampal dentate gyrus of OXYS rats. Delayed neuronal maturation led to alterations of mossy-fiber formation: a shortened suprapyramidal bundle and longer infrapyramidal bundle, less pronounced fasciculation of granule cells’ axons, and smaller size and irregular shape of nuclei in the CA3 pyramidal layer. These changes were accompanied by altered astrocytic migration. The observed features of early development may be considered some of the risk factors of the AD-like pathology that manifests itself in OXYS rats late in life.

## Introduction

Alzheimer’s disease (AD) is detrimental neurodegenerative disorder that over 50 million people suffer from, and a figure set to increase to 152 million by 2050 (Alzheimer’s Disease International, 2019). Age is the most significant risk factor for the sporadic form of AD accounting for more than 95% of cases and characterized by cognitive deficit manifesting after the age of 65 years on the background of irreversible neurodegenerative changes and develops asymptomatically for many years prior to its manifestation (Hersi et al., 2017). At present, it is increasingly recognized that middle age is the most critical period for the onset of AD and is followed by asymptomatic disease development for many years prior to its manifestation (Bateman et al., 2012; Crous-Bou et al., 2017). Genetic and epigenetic factors have a substantial impact on the age of disease manifestation and the rate of its progression, while comorbidities might accelerate neurodegeneration and the development of dementia (Morris et al., 2014). Moreover, to date, several experimental and epidemiological studies have shown that risk factors for the development of AD may materialize early in life: during postnatal brain maturation and formation of synapses. One of such risk factors is the formation of aberrant neural circuits under the influence of genetic and/or environmental factors (Kovács et al., 2014; Axelrud et al., 2019). Recently, it was reported that prenatal hypoxia (Nalivaeva et al., 2018) and low weight at birth (Heinonen et al., 2015) may be among these factors determining the brain development trajectory as well as the risk of AD. Such conditions can be caused by preterm birth (including 1st degree preterm birth, which usually is not accompanied by cognitive deficits) as well as by trophic insufficiency during gestation owing to a number of causes (Lesuis et al., 2018). Another important factor which may exert long-lasting effects on brain functioning and contribute to neurodegeneration is perinatal infections. Indeed, because of early migration of microglia to developing brain perinatal infections lead to microglial activation, which in turn results in inhibition of axonal growth and collapsing of axon growth cone (Reemst et al., 2016; Sominsky et al., 2018). However, the mechanisms and substrates underlying these long-lasting effects remain unclear. Indeed, in humans, it is difficult to establish this connection because of the long period between the events of early life and the emergence of clinical symptoms. Even though the time scales are considerably different, the sequence of key events of brain maturation is largely consistent between humans and rodents, however, experimental research is constrained by the lack of valid biological models of the sporadic form of such a complex condition as AD.

In recent years, convincing arguments were made confirming that the strain of senescence-accelerated OXYS rats meets the requirements for the AD model owing spontaneously develop all the major signs of AD and largely reproduce the stages of the disease (Stefanova et al., 2014, 2018, 2019). Indeed, structural neurodegenerative alterations, synaptic damage and neuronal loss at the background of mitochondrial dysfunction, accumulation of amyloid-β and tau hyperphosphorylation in the hippocampus, cerebrovascular alterations and cognitive impairment observed in OXYS rats. It is important to emphasize that these features develop spontaneously without mutations in *App*, *Psen1*, and *Psen2* genes in OXYS rats, and the course of these changes matches sporadic AD development in humans. However, the sequence of events leading to development of AD-like pathology in OXYS rats is still unknown. More recently, we demonstrated that alterations of neurogenesis accompany the development of AD-like pathology in OXYS rats (Rudnitskaya et al., 2019). We showed that the delay of the peak of neuronal density and of apoptosis in the hippocampus of OXYS rats is accompanied by retardation of postnatal reflex development, possibly implying a slowing of postnatal neurogenesis and alteration of mossy-fiber formation in the dentate gyrus (DG) of the hippocampus in OXYS rats. We hypothesized that the features of early hippocampal development may be regarded as one of the risk factors of AD-like pathology in OXYS rats. To verify this supposition, in this study, we evaluated the duration of pregnancy and brain parameters reflecting brain maturity at birth and in the period of postnatal development (e.g., the magnitude of neurogenesis, formation of mossy fibers, and astrocytic support of the neurogenic niche in the hippocampus) as well as the behavior of OXYS puppies compared to the control (Wistar) rat strain.

## Materials and Methods

### Animals

Senescence-accelerated OXYS rats and age-matched Wistar rats were obtained from the Breeding Experimental Animal Laboratory of the Institute of Cytology and Genetics, SB RAS, Novosibirsk, Russia. The OXYS strain was derived from the Wistar strain of rats at the Institute of Cytology and Genetics as described earlier (Stefanova et al., 2010) and was registered in the Rat Genome Database.^1^ At this point, we have the 112th generation of OXYS rats, with spontaneously developing cataract and accelerated senescence syndrome inherited in a linked manner. The animals were kept under standard laboratory conditions (22°C ± 2°C, 60% relative humidity, and 12 h light/12 h dark cycle) and had *ad libitum* access to standard rodent feed (PK-120-1, Laboratorsnab, Ltd., Russia) and water.

### Reproductive Parameters and Maternal Data

Sexually naïve 3-month-old female rats (*n* = 20 per group) were weighed and then mated with age-matched males. Pregnancy was identified by the presence of spermatozoa in vaginal smears the following morning, which was designated gestational day 0. We assessed the duration of gestation, litter size, and the sex ratio of the pups as well as body weight, brain weight, and the brain-to-body weight ratio [meaning (brain weight ÷ body weight) × 100%] of male pups on postnatal day 0 (PND0), PND10, PND14, PND20, and PND45.

### Behavioral Testing

We evaluated locomotor activity and anxiety of male rats by the open field test and elevated plus maze test. Each test was performed once per animal. The test sessions were scheduled between 10 a.m. and 2 p.m.

#### The Open Field Test

The test was conducted to estimate locomotor and exploratory activity of OXYS and Wistar rats at PND20 and PND45 (*n* = 20 per group). The open-field area consisted of an enclosed square arena made of opaque Plexiglas (100 × 100 cm) surrounded by walls (40 cm high). The arena was divided by transverse lines into 100 equal squares. A central area was arbitrarily defined as a square of 40 × 40 cm. A central light source (100 W) on the ceiling provided invariant illumination in an otherwise dark room. Each rat was placed into the same corner of the arena facing in the same direction and was allowed to freely explore the arena for 300 s. Every time both hind limbs entered a square, a crossing was recorded. The locomotor and exploratory activities were evaluated by counting the line crossings and then converting this parameter to the distance passed as well as by counting rearing events (when an animal stood on its hind limbs). In addition, anxiety was evaluated by recording the time before the first entry into the central area. A rat was assumed to be in the central area when its four limbs were on it. The number of self-grooming actions was determined too.

To evaluate the locomotor activity of OXYS and Wistar rats at PND10 and PND14 (*n* = 20 per group), we modified the arena of the open field as well as duration of each session in accordance with the Shimomura and Ohta (1988). The area consisted of an enclosed rectangular arena (20 × 30 cm) divided by transverse lines into 24 equal squares (5 × 5 cm). Each animal was allowed to freely explore the arena for 150 s. The distance passed, the number of forelimb lifts, and the number of self-groomings was determined.

#### The Elevated Plus Maze Test

Anxiety of OXYS and Wistar rats at PND45 (*n* = 20 per group) was estimated using the elevated plus maze test. The plus maze apparatus was made of opaque Plexiglas and contained two opposite open arms (50 × 10 cm) and two closed arms of the same size but with 40-cm-high walls. Each arm was divided by lines into five equal squares (10 × 10 cm). The apparatus was elevated 50 cm above the floor. Each rat was placed in the central square of the plus maze, facing one of the closed arms, and its behavior was analyzed for 300 s. To analyze animal behavior in the elevated plus maze test, we recorded the criteria described by Rodgers and Dalvi (1997).

### Tissue Preparation

Animals were euthanized by CO_2_ asphyxiation and decapitated; the brains were carefully removed, and hemispheres were separated. For an immunohistochemical assay, the right hemisphere was immediately fixed in 4% paraformaldehyde in phosphate-buffered saline (PBS) at room temperature (RT) for 48 h followed by cryoprotection in 30% sucrose in PBS at 4°C for 48 h. Then, the brains were frozen and stored at −70°C until further processing.

### Immunohistochemistry

Brain sagittal sections (20 μm thick) of OXYS and Wistar rats at PND10, PND14, PND20, and PND45 [*n* = 3 to 6 animals per strain (genotype) and age; 2–3 slices per animal] were prepared on a Microm HM-505 N cryostat (Microm, Germany) at −20°C and transferred onto polysine-glass slides (Menzel-Glaser, Braunschweig, Germany). After serial washes with PBS, the slices were incubated at RT for 15 min in PBS-plus (PBS with 0.1% of Triton X-100) and for 1 h in 3% bovine serum albumin (BSA; cat. # A3294, Sigma-Aldrich, United States) in PBS to permeabilize the tissues and to block nonspecific binding sites, and then were incubated overnight with primary antibodies at 4°C. The primary antibodies were all diluted 1:250 with 3% BSA in PBS: these were antibodies to Ki67, nestin, vimentin, and glial fibrillary acid protein (GFAP) (cat. ## ab15580, ab6142, ab24525, and ab7260, respectively, Abcam, United States). After several washes with PBS, the slices were incubated with secondary antibodies conjugated with Alexa Fluor 488, 568, or 555 (cat. ## ab150073, ab175472, and ab150170, respectively, Abcam, United States) in PBS (1:250) for 1 h at RT and then were washed in PBS. The slices were coverslipped with the Fluoroshield mounting medium containing 4′,6-diamidino-2-phenylindole (DAPI; cat. # ab104139, Abcam, United States). Negative controls were processed in an identical manner except that a primary antibody was not included. The Ki67, nestin, vimentin, and GFAP signals were counted under a microscope with a 40× objective lens (Axioskop 2 plus, Zeiss, Germany). The microscopy was conducted at the Multi-Access Center for Microscopy of Biological Objects (Institute of Cytology and Genetics, SB RAS, Novosibirsk, Russia). Identification of cell types was conducted according to protein markers described by Encinas et al. (2011). To assess the density of proliferating (Ki67-positive) cells, quiescent (nestin-positive and vimentin-positive) and amplifying (nestin-positive) neural progenitors as well as astrocyte progenitors (vimentin-positive and GFAP-positive) and astrocytes (GFAP-positive), the total number of counted cells was divided by the area of the DG and was presented as the number of cells per 10000 μm^2^.

### Visualization of Mossy Fibers

Hippocampi were excised from the brains of OXYS and Wistar rats at PND0, PND10, PND14, PND21, and PND45 (*n* = 6 to 8 animals per genotype and age), transversely sliced at a thickness of 400 μm and fixed in 4% paraformaldehyde at +4°C for 24 h. Mostly, the slices from dorsal and medium parts of the hippocampus were analyzed. To evaluate morphological characteristics of mossy fibers, crystals of 1,1′-dioctadecyl-3,3,3′,3′-tetramethylindocarbocyanine perchlorate (DiI; Molecular Probes, United States) were diluted in a dimethyl sulfoxide and ethanol mixture (2:1); then the resultant solution (2 mg/ml) was injected into the DG area of hippocampal slices. The slices were incubated in PBS for 7 days necessary for dye spreading from neuronal somata to the axons. To reveal cell layers, the slices were additionally stained with Hoechst 33342 stain (Sigma-Aldrich, United States) at 3 μg/ml for 20 h. The analysis was conducted under a confocal microscope (LSM-780-NLO, Zeiss, Germany) at the Multi-Access Center for Microscopy of Biological Objects (the Institute of Cytology and Genetics, SB RAS, Novosibirsk, Russia). Morphometric measurements were carried out in standard microscopic software on z projections of five consecutive confocal images of each slice usually at a depth of 15–25 μm.

**FIGURE 1:**
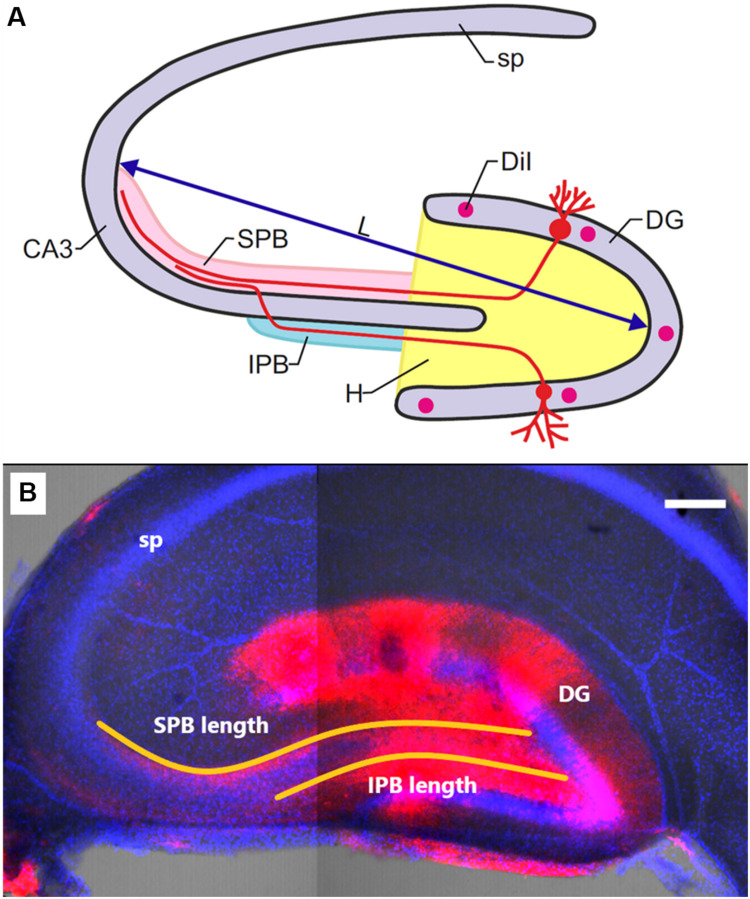
**(A)** Schematic illustration of mossy-fiber location in a transverse section of the rat hippocampus. DG: dentate gyrus, sp: stratum pyramidale, CA3: hippocampal area CA3, H: hilus, SPB and IPB: suprapyramidal and infrapyramidal bundles of mossy fibers, DiI: injection sites of 1,1′-dioctadecyl-3,3,3′3′-tetramethylindocarbocyanine perchlorate, L: maximal distance between the DG and CA3 used for determining slice size. **(B)** Representative image of the hippocampus of OXYS rat at PND10. DiI (red) labels mossy fibers; Hoechst (blue) indicates cell nuclei. The scale bar is 200 μm.

Figure 1 shows the positions of cell layers (DG and CA3), of the suprapyramidal bundle (SPB) and infrapyramidal bundle (IPB), as well as the sites of DiI injection. Age-dependent changes of slice size were evaluated as a distance between maximally distant points of the granule cell layer and pyramidal neurons of CA3 (L); relative lengths of the IPB and SPB were calculated as IPB/L and SPB/L ratios. The width of the bundles was measured in the area where the bundles leave the hilus. The width of the pyramidal layer was evaluated according to location of stained pyramidal cells’ nuclei in the area adjacent to the hilus.

### Statistics

The data were subjected to two-way analysis of variance (ANOVA) in the Statistica 8.0 software (StatSoft, United States). The genotype, age, and sex were chosen as independent variables. The Newman–Keuls *post hoc* test was applied to significant main effects and interactions in order to assess the differences between some sets of means. The data were presented as mean ± standard error of the mean (SEM). The differences were considered statistically significant at *p* < 0.05.

## Results

### Duration of Gestation and Litter Size

In OXYS rats, the duration of gestation was shorter than that in Wistar rats (*p* < 0.0003; Table 1) and could cause a delay in physical development of the pups. In addition, litter size was smaller in OXYS rats (*p* < 0.001) with a decreased number of male (*p* < 0.0001) and female pups (*p* < 0.002), whereas the male/female ratio remained unchanged (Table 1). It should be mentioned that the body weight of adult male OXYS rats is lower as compared to Wistar rats (Kolosova et al., 2011). Here we showed that the body weight of 3-month-old female OXYS rats is also lower relative to Wistar rats (*p* < 0.007; Table 1); this difference may explain the decreased litter size in OXYS rats. Nonetheless, our analysis did not detect any correlations between female body weight and litter size (*r* = −0.19, *p* = 0.68). Thus, the decreased female body weight is not the leading cause of the smaller litter size in OXYS rats.

**TABLE 1:** Duration of gestation and litter size of OXYS and Wistar rats.

Parameter	Wistar rats	OXYS rats
Maternal body weight, g	351 ± 19	274 ± 10*
Duration of gestation, days	23.2 ± 0.17	22.4 ± 0.12*
Litter size	13.8 ± 0.15	11.2 ± 0.14*
Number of male pups	7.2 ± 0.24	5.8 ± 0.23*
Number of female pups	7.3 ± 0.19	6.4 ± 0.21*
Ratio male/female pups	1.14 ± 0.12	0.94 ± 0.10

The data are presented as mean ± SEM; *p < 0.05 for differences between strains.

### The Brain-to-Body Weight Ratio

Body weight naturally increased with age (*F*_4,106_ = 2685.3, *p* < 0.0001) in OXYS and Wistar rats and was affected by the genotype (*F*_1,106_ = 90.2, *p* < 0.0001) because at birth (PND0) and at PND45, body weight was lower in OXYS rats than in Wistar rats (Table 2).

**TABLE 2:** Body and brain weight and the brain-to-body weight ratio in OXYS and Wistar male pups.

Postnatal day	Wistar rats			OXYS rats		
	Body weight, g	Brain weight, g	Brain-to-body weight ratio, %	Body weight, g	Brain weight, g	Brain-to-body weight ratio, %
PND0	6.08 ± 0.36	0.291 ± 0.006	4.96 ± 0.30	4.17 ± 0.27*	0.238 ± 0.010*	6.01 ± 0.50
PND10	20.19 ± 0.79^#^	0.984 ± 0.015^#^	4.95 ± 0.15	19.75 ± 0.86^#^	1.044 ± 0.016*^#^	5.37 ± 0.18
PND14	32.31 ± 1.56^#^	1.274 ± 0.018^#^	4.03 ± 0.12^#^	29.64 ± 1.01^#^	1.299 ± 0.019^#^	4.44 ± 0.14*^#^
PND20	45.58 ± 1.59^#^	1.421 ± 0.013^#^	3.16 ± 0.11^#^	45.33 ± 1.46^#^	1.437 ± 0.011^#^	3.20 ± 0.10^#^
PND45	197.2 ± 3.99^#^	1.654 ± 0.035^#^	0.84 ± 0.01^#^	158.0 ± 3.86*^#^	1.636 ± 0.032^#^	1.04 ± 0.01*^#^

The data are presented as mean ± SEM; *p < 0.05 for differences from Wistar rats; ^#^*p* < 0.05 for differences from a previous age.

Weight of the brain also naturally increased with age (*F*_4,106_ = 1854.0, *p* < 0.0001) and was not affected by the genotype (*F*_1,106_ = 0.3, *p* = 0.60). Nevertheless, these factors interacted (*F*_4,106_ = 3.5, *p* < 0.01): in OXYS rats at PND0, the weight of the brain was lower (*p* < 0.0002) and at PND10 it was higher (*p* < 0.01) when compared to Wistar rats (Table 2).

The brain-to-body weight ratio (Table 2) was affected by the genotype (*F*_1,106_ = 7.3, *p* < 0.008) and age (*F*_4,106_ = 80.6, *p* < 0.0001). In both Wistar and OXYS rats, the brain-to-body weight ratio remained unchanged from PND0 to PND10 and decreased until PND14 (*p* < 0.0005) with a further decrease until PND20 (*p* < 0.0001) and PND45 (*p* < 0.0001). At the same time, the brain-to-body weight ratio was higher in OXYS rats than in Wistar rats at PND45 (*p* < 0.0001) because of the decreased body weight, as was the case at PND14 (*p* < 0.04).

Taken together, these results indicated specific features of the dynamics of the brain-to-body weight ratio in OXYS rats.

### Behavioral Features of OXYS Rats at the Age of Brain Maturation

Because of its complexity, animal behavior reflects alteration of processes occurring in the brain. To evaluate the functionality of the brain during its maturation, we studied locomotor activity of OXYS and Wistar rats at PND10, PND14, PND20, and PND45.

#### Locomotor Activity of the Animals in the Open Field Test

Because of various durations of the open field test for animals of different ages, we compared the distance passed by rats during 150 s at PND10 and PND14 and during 300 s at PND20 and PND45.

**FIGURE 2:**
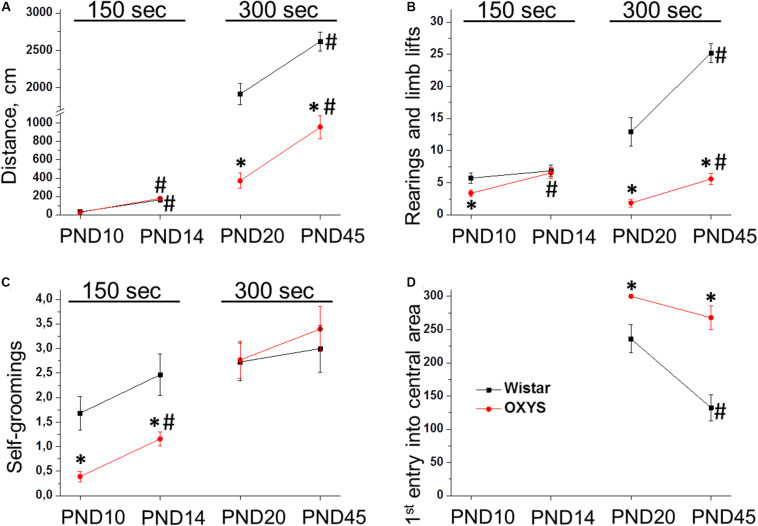
Locomotor activity of OXYS and Wistar rats at PND10, PND14, PND20, and PND45 in the open field test. The distance passed **(A)** and the number of rearings **(B)** increased with age in both rat strains, however, they were lower in OXYS rats at PND20 and PND45. The frequency of self-grooming actions **(C)** was lower in OXYS rats at PND10 and PND14. The time before the first entry into the central area **(D)** was longer in OXYS rats, thereby reflecting increased anxiety. The data are presented as mean ± SEM; *p < 0.05 for differences from Wistar rats; ^#^*p* < 0.05 for differences from a previous age.

We found that the distance passed by the animals (Figure 2A) significantly increased with age (*F*_1,75_ = 98.2, *p* < 0.0001 for PND10 and PND14; *F*_1,60_ = 149.5, *p* < 0.0001 for PND20 and PND45); whereas at PND20 and PND45, the distance passed by OXYS rats was lower in comparison with Wistar rats (*F*_1,60_ = 24.0, *p* < 0.0001).

As for the number of forelimb lifts (Figure 2B), it increased from PND10 to PND14 (*F*_1,75_ = 8.0, *p* < 0.006). In OXYS rats, this parameter was lower at PND10 (*p* < 0.02) and reached the level of Wistar rats at PND14. The number of rearings (Figure 2B) increased from PND20 to PND45 (*F*_1,60_ = 33.3, *p* < 0.0001) and was lower in OXYS rats (*F*_1,60_ = 121.6, *p* < 0.0001).

The frequency of self-groomings (Figure 2C) increased from PND10 to PND14 (*F*_1,75_ = 8.5, *p* < 0.005) and was lower in OXYS rats (*F*_1,75_ = 23.7, *p* < 0.0001). The lower frequency of self-grooming behavior observed in OXYS rats may reflect decreased anxiety and/or decreased locomotor activity of the animals. We did not observe effects of the genotype (*F*_1,60_ = 0.2, *p* = 0.65) or age (*F*_1,60_ = 0.9, *p* = 0.35) on the frequency of self-groomings at PND20 and PND45 (Figure 2C).

In addition, for 20- and 45-day-old rats, we recorded the time before the first entry into the central area reflecting the anxiety level of the animals (Figure 2D). We demonstrated that this parameter decreased with age (*F*_1,60_ = 13.1, *p* < 0.0006) and was higher in OXYS rats (*F*_1,60_ = 28.2, *p* < 0.0001). On the other hand, *post hoc* analysis showed that the parameter did not statistically significantly differ between PND20 and PND45 in OXYS rats (*p* > 0.05). The absence of a decrease in the time before the first entry into the central area in OXYS rats together with a significant increase in the passed distance from PND20 to PND45 may indicate increased anxiety of OXYS rats at PND45.

Taken together, these results meant a significant increase in the locomotor activity of rats from PND10 to PND45. Nevertheless, at younger ages, locomotor activity did not differ between OXYS and Wistar rats, but we observed significantly lower locomotor activity in OXYS rats than in Wistar rats at PND20 and PND45. Furthermore, we noted signs of increased anxiety in OXYS rats at PND45; therefore, next, we analyzed the anxiety of Wistar and OXYS rats at PND45 by the elevated plus maze test.

#### The Level of Anxiety at PND45 in the Elevated Plus Maze Test

**FIGURE 3:**
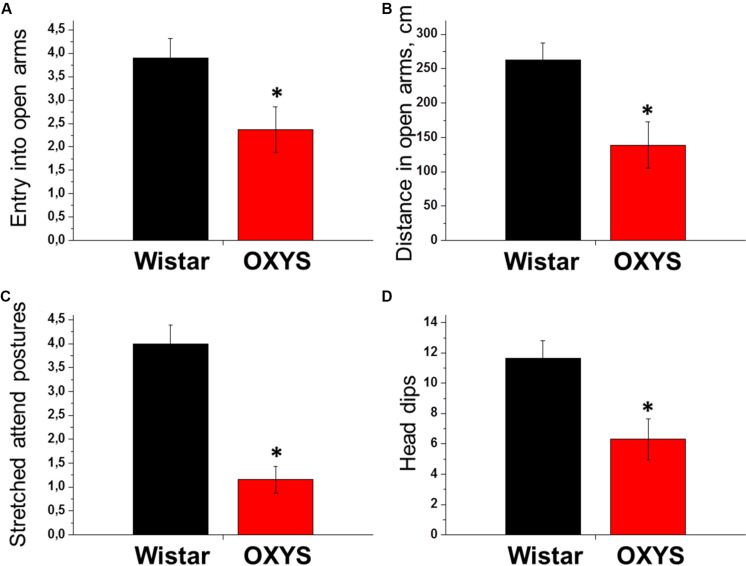
Examination of rat behavior in the elevated plus maze test at PND45. The number of entries into the open arms of the maze **(A)** and the distance passed in them **(B)** reflect the locomotion and anxiety levels of animals: more anxious animals make fewer entries into open arms and pass shorter distance in it compared to less anxious animals. The numbers of stretched attend postures from close to open arm **(C)** and head dips from open arm **(D)** reflect exploratory activity and anxiety level of animals: more anxious animals make fewer stretched attend postures and head dips compared to less anxious animals. All these parameters were lower in OXYS rats, thus reflecting decreased locomotion and exploratory activity and increased anxiety. The data are presented as mean ± SEM; *p < 0.05 for differences from Wistar rats.

The number of entries into the open arms and squares crossed in them by OXYS rats at PND45 were 1.5- and 2-fold lower relative to age-matched Wistar rats (*p* < 0.03 and *p* < 0.006, respectively, Figures 3A,B). It may be because of decreased locomotion of OXYS rats and/or point out to increased level of anxiety. Moreover, the number of entries into the closed arms and squares crossed in them as well as the frequency of rearings were also lower in OXYS rats (*p* < 0.001, *p* < 0.0001, and *p* < 0.0001, respectively) reflecting decreased locomotor activity. In addition, we registered a decrease in the exploratory activity of OXYS rats: the numbers of stretched attend postures and head dips were lower as compared to Wistar rats (*p* < 0.0001 and *p* < 0.005, respectively, Figures 3C,D).

Therefore, OXYS rats exhibited a decrease in locomotor and exploratory activities and signs of increased anxiety already at a young age. The progression of these behavioral alterations may reflect perturbations of hippocampal neurogenesis during brain maturation.

### Hippocampal Neurogenesis During Brain Maturation in OXYS and Wistar Rats

**FIGURE 4:**
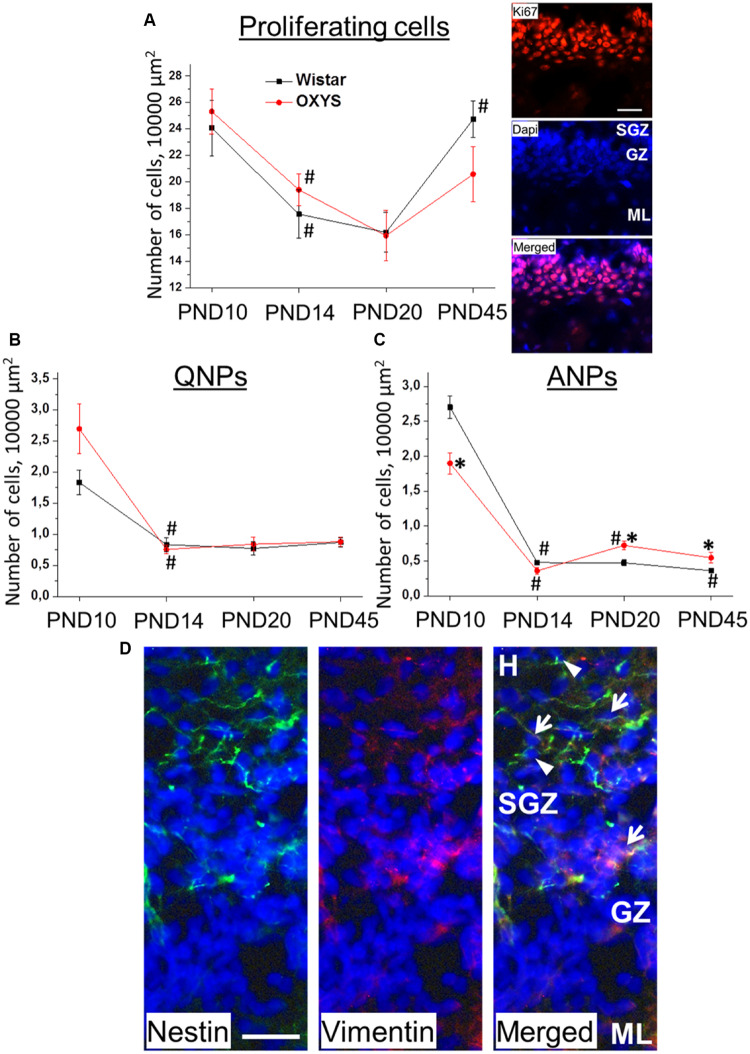
Densities of proliferating cells, QNPs and ANPs, in the DG of OXYS and Wistar rats at different stages of postnatal development. The density of proliferating cells **(A)** decreased from PND10 to PND20 in both rat strains and increased until PND45 in Wistar rats; photomicrograph of the DG of OXYS rat at PND10 was used as representative image of immunohistochemical staining with antibodies against Ki67 (red); DAPI (blue) indicates cell nuclei. Densities of QNPs **(B)** and ANPs **(C)** decreased by PND14 in both rat strains; ANP density was higher in OXYS rats at PND20 and PND45. The data are presented as mean ± SEM; *p < 0.05 for differences from Wistar rats; ^#^*p* < 0.05 for differences from a previous age. **(D)** Photomicrograph of the DG of OXYS rat at PND10 was used as representative image of immunohistochemical staining with antibodies against nestin (green) and vimentin (red). DAPI (blue) indicates cell nuclei. Arrows show QNPs, and arrowheads show ANPs. The scale bar **(A,D)** is 30 μm. H, hilus; SGZ, subgranule zone; GZ, granule zone; ML, molecular layer of DG.

We found that the density of proliferating cells was affected only by age (*F*_3,63_ = 9.2, *p* < 0.0001; Figure 4A): this density decreased from PND10 to PND14 in Wistar and OXYS rats (*p* < 0.03 and *p* < 0.009, respectively), did not statistically significantly change from PND14 to PND20 in both rat strains, and increased from PND20 to PND45 only in Wistar rats (*p* < 0.001).

The density of quiescent neural progenitors (QNPs) naturally decreased with age (*F*_3,69_ = 34.6, *p* < 0.0001; Figure 4B). *Post hoc* analysis revealed a tendency of QNP density to be higher in OXYS rats than in Wistar rats at PND10 (*p* = 0.073).

The density of amplifying neural progenitors (ANPs) was affected by age (*F*_3,69_ = 216.0, *p* < 0.00001) and weakly affected by genotype (*F*_1,69_ = 4.0, *p* = 0.05). Furthermore, we noted different effects of age on the different rat strains (*F*_3,69_ = 14.8, *p* < 0.0001; Figure 4C): in Wistar rats, ANP density decreased from PND10 to PND14 (*p* < 0.0001) and then from PND20 to PND45 (*p* < 0.04), whereas in OXYS rats, it was lower at PND10 (*p* < 0.003), decreased to PND14 (*p* < 0.0001) remaining lower than in Wistar rats with borderline significance (*p* = 0.06), and then increased until PND20 (*p* < 0.0001) becoming higher than in Wistar rats (*p* < 0.005) and remaining at the increased level at PND45 (*p* < 0.03). Photomicrograph of QNPs and ANPs in DG is presented in the Figure 4D.

In order to assess the relationship between the duration of pregnancy and neurogenesis, we conducted a correlation analysis between duration of gestation and densities of proliferating and progenitor cells. We revealed significant negative correlation between duration of gestation and density of quiescent neural progenitors at PND10 (*r* = −0.36, *p* < 0.04); besides we observed a tendency for negative correlation between duration of gestation and density of proliferating cells (*r* = −0.33, *p* = 0.06) and for positive correlation between duration of gestation and density of amplifying neural progenitors (*r* = 0.31, *p* = 0.08). However, even for QNPs, the correlation was statistically significant but not strong.

Thus, the density of proliferating cells and progenitors in the DG of Wistar and OXYS rats naturally decreased until PND14, thereby reflecting deceleration of postnatal neurogenesis and the onset of hippocampal maturation. Nonetheless, the decrease of ANP density at PND10 and its increase at PND20 and PND45 in OXYS rats may point to retardation of the postnatal peak of neurogenesis in the hippocampus.

### Morphological Characteristics of Mossy Fibers in OXYS and Wistar Rats

**FIGURE 5:**
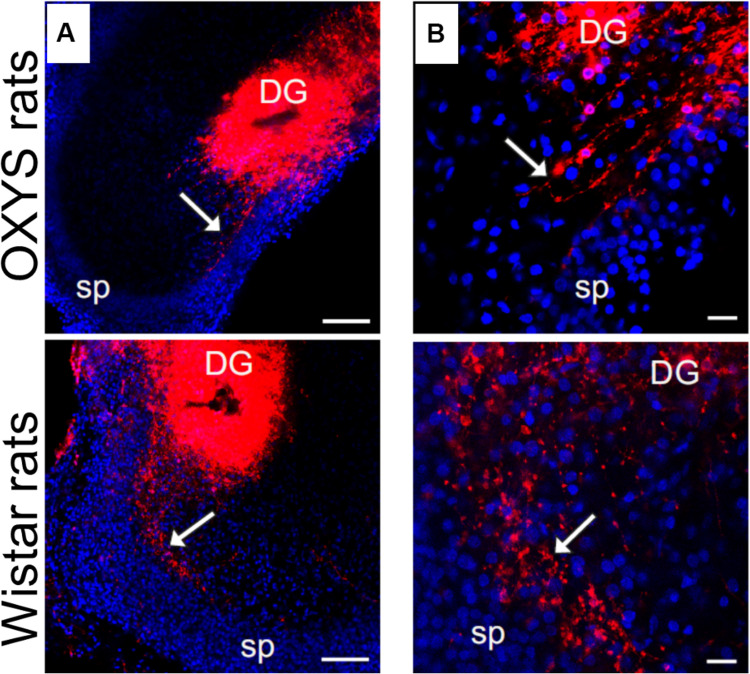
Developing mossy fibers in hippocampal slices from newborn OXYS and Wistar rats. Confocal images of DiI-labeled granular-cell axons. The SPB (indicated by the arrow) is already formed in Wistar rats’ hippocampal slices, while only a few axons can be seen in OXYS rats. The IPB is not detectable yet. **(A)** Hippocampal slices at low magnification; **(B)** the bundle of mossy fibers at the border of the hilus and CA3. DiI (red) labels mossy fibers; Hoechst (blue) indicates cell nuclei. Scale bars are 100 μm **(A)** and 20 μm **(B)**; sp: stratum pyramidale.

Development of mossy fibers in hippocampal slices from newborn OXYS and Wistar rats is illustrated in Figure 5. The figure shows that at PND0, the SPB already formed in Wistar rats’ hippocampal slices, whereas only a few axons were seen in OXYS rats, thus reflecting deterioration of SPB formation. The IPB was not detected at PND0 in either rat strain.

**FIGURE 6:**
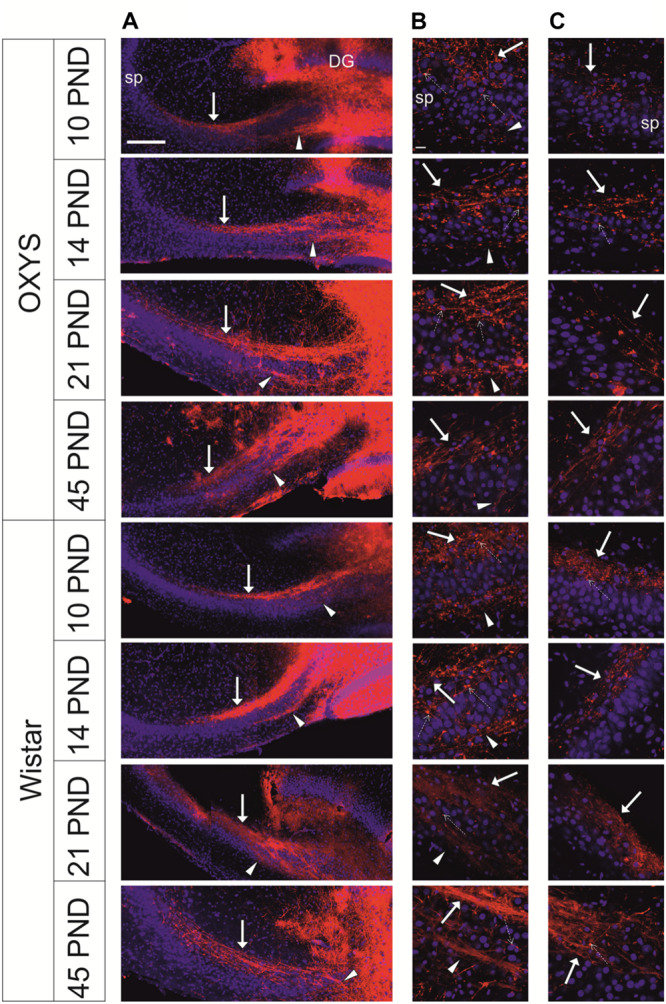
Representative confocal images of mossy-fiber projections in hippocampal slices from OXYS and Wistar rats at different stages of postnatal development. **(A)** Hippocampal slices at low magnification; **(B)** the SPB at the border of the hilus and CA3; **(C)** the distal end of the SPB at the border of CA3 and CA2. DiI (red) labels mossy fibers; Hoechst (blue) indicates cell nuclei. Solid arrows show the SPB, arrowheads show the IPB, and dashed arrows show varicosities. Scale bars are 200 μm **(A)** and 20 μm **(B,C)**; sp: stratum pyramidale.

We did not observe any alterations of the location of the IPB and SPB in Wistar rats at all the examined ages. Meanwhile, in OXYS rats, the density of fibers in the SPB and IPB was lower, and the bundles were less compact likely because of decreased fasciculation of granule cells’ axons (Figure 6). We detected the presence of small and large varicosities along several fibers; large varicosities are usually considered developing mossy synapses. Fewer large varicosities were observed in OXYS rats than in Wistar rats (Figure 6), however, the time of emergence of varicosities did not differ between the rat strains. As for the CA3 pyramidal layer, in OXYS rats, neuronal nuclei were smaller and were frequently characterized by an irregular shape (Figure 6).

**FIGURE 7:**
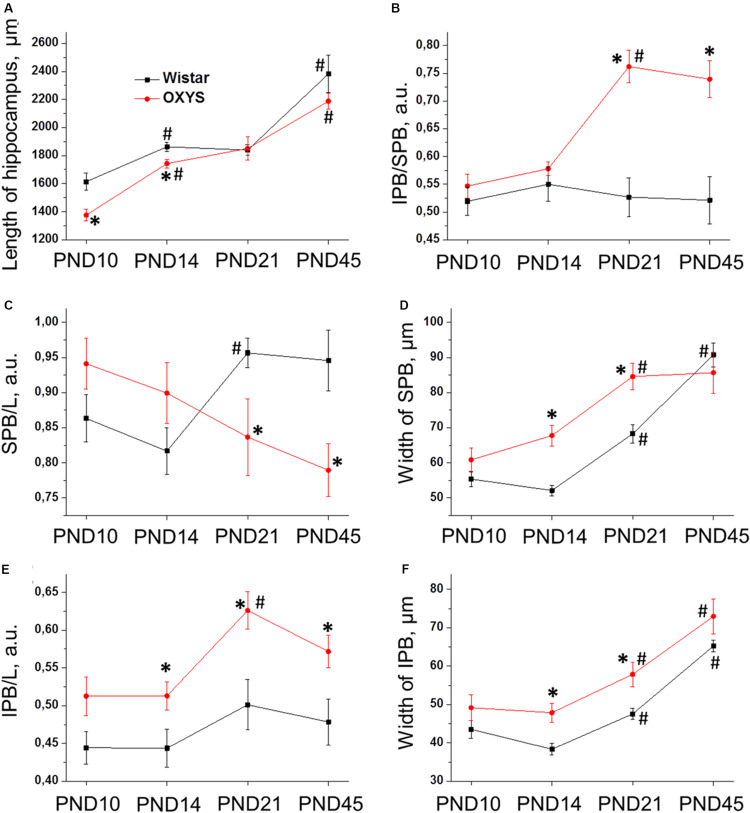
Morphometric measurements of confocal images of mossy-fiber projections in hippocampal slices from OXYS and Wistar rats at different stages of postnatal development. **(A)** The maximal distance between the DG and CA3 (L); **(B)** the ratio of IPB length to SPB length of mossy fibers in the hippocampus; **(C)** normalized length of the SPB; **(D)** width of the SPB; **(E)** normalized length of the IPB; **(F)** width of the IPB. The data are presented as mean ± SEM; *p < 0.05 for differences from Wistar rats; ^#^*p* < 0.05 for differences from a previous age.

We found that the longitudinal size of slices (L) (Figure 7A) naturally increased with age (*F*_3,101_ = 55.2, *p* < 0.0001), and this parameter was lower in OXYS rats (*F*_1,101_ = 9.2, *p* < 0.003). By contrast, by PND21, these differences disappeared, and at PND21 and PND45, L did not differ between OXYS and Wistar rats.

Regarding pyramidal-layer width, we detected significant effects of the genotype (*F*_1,101_ = 5.7, *p* < 0.02) and age (*F*_3,101_ = 17.4, *p* < 0.0001) as well as an interaction between these factors (*F*_3,101_ = 4.7, *p* < 0.004). In Wistar rats, this value decreased from PND10 to PND14 (*p* < 0.006) and then gradually increased by PND45 (*p* < 0.0001), whereas in OXYS rats, the decrease in pyramidal-layer thickness from PND10 to PND14 was statistically insignificant (*p* > 0.05), and thus this parameter became higher relative to Wistar rats (*p* < 0.02), then the width of the pyramidal layer increased until PND21 (*p* < 0.0001) and became higher as compared to Wistar rats (*p* < 0.0002).

Next, we analyzed morphological characteristics of the SPB and IPB. Normalized length of the SPB (the SPB/L ratio) was not affected by the genotype (*F*_1,101_ = 1.1, *p* = 0.30) or age (*F*_3,101_ = 0.6, *p* = 0.59), however, there was an interaction between these factors (*F*_3,101_ = 5.4, *p* < 0.002). We observed differences in age-related changes of this parameter between Wistar and OXYS rats (Figure 7C): in Wistar rats, the SPB/L ratio did not change from PND10 to PND14, increased by PND21, and remained at the same level until PND45, whereas in OXYS rats, this parameter gradually decreased from PND10 to PND45 (*p* < 0.004) becoming lower than in Wistar rats at PND21 and PND45 (*p* < 0.03 and *p* < 0.01, respectively). SPB width (Figure 7D) increased with age (*F*_3,101_ = 32.6, *p* < 0.0001) and was greater in OXYS rats (*F*_1,101_ = 9.9, *p* < 0.002). In particular, the greatest increase of SPB width in Wistar rats occurred from PND14 to PND45, whereas in OXYS rats, it changed from PND10 to PND21; finally, at PND45, there were no statistically significant differences in SPB width between the two rat strains.

As to the IPB, we found that normalized length of the IPB (the IPB/L ratio; Figure 7E) and its thickness (Figure 7F) increased with age (*F*_3,101_ = 4.9, *p* < 0.003 and *F*_3,101_ = 33.7, *p* < 0.0001, respectively) and were higher in OXYS rats (*F*_1,101_ = 24.3, *p* < 0.0001 and *F*_1,101_ = 16.1, *p* < 0.0002, respectively).

The IPB/SPB ratio may be regarded as an indicator of mossy-fiber maturation. We observed significant effects of the genotype (*F*_1,101_ = 37.0, *p* < 0.0001) and age (*F*_3,101_ = 6.3, *p* < 0.0006) on this parameter as well as an interaction between these factors (*F*_3,101_ = 7.6, *p* < 0.0001). We did not detect significant changes of the IPB/SPB ratio in Wistar rats, but this was not the case in OXYS rats (Figure 7B): indeed, in OXYS rats, the parameter significantly increased from PND14 to PND21 (*p* < 0.0001)—thus becoming higher than in Wistar rats (*p* < 0.0001)—and remained at the same level until PND45.

Consequently, we revealed the following features of mossy-fiber formation in OXYS rats: a smaller SPB and lager IPB, less pronounced fasciculation of granule cell axons, and smaller size and an irregular shape of nuclei in the CA3 pyramidal layer.

### Astrocytic Support of the Hippocampal Neurogenic Niche During Brain Maturation in OXYS and Wistar Rats

**FIGURE 8:**
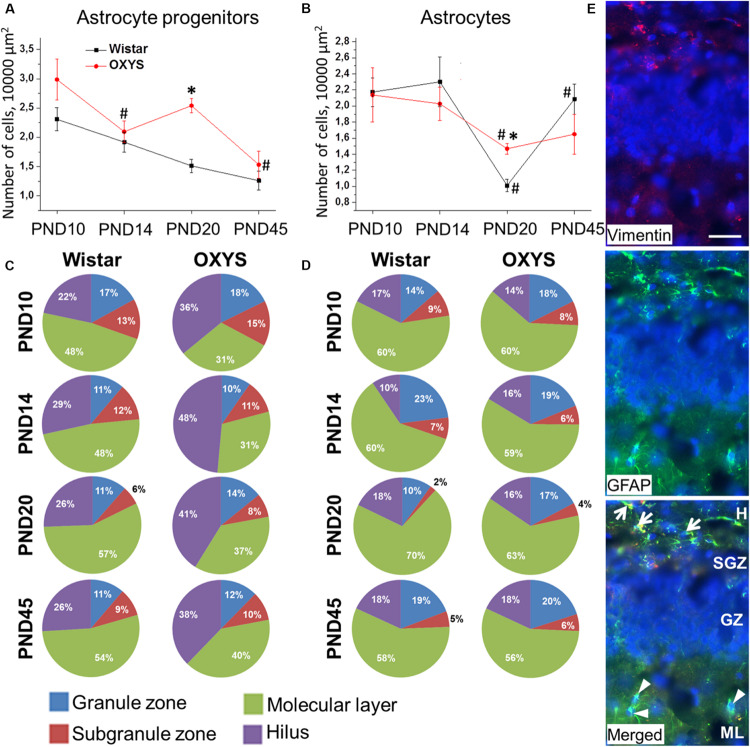
Density and distribution of astrocytic cells and their progenitors in the DG of OXYS and Wistar rats at different stages of postnatal development. The densities of astrocytes **(B)** and their progenitors **(A)** were higher in OXYS rats at PND20. The distribution of astrocytes **(D)** did not differ between OXYS and Wistar rats, whereas the percentage of astrocyte progenitors **(C)** was lower in the molecular layer and higher in the hilus of OXYS rats. The data are presented as mean ± SEM; *p < 0.05 for differences from Wistar rats; ^#^*p* < 0.05 for differences from a previous age. **(E)** Photomicrograph of the DG of OXYS rat at PND10 was used as representative image of immunohistochemical staining with antibodies against GFAP (green) and vimentin (red). DAPI (blue) indicates cell nuclei. The scale bar is 30 μm. Arrows show astrocyte progenitors, and arrowheads show astrocytes. H, hilus; SGZ, suggranular zone; GZ, granule zone; ML, molecular layer of DG.

Astrocytic support is crucial for proper functioning of neurogenic niches. ANOVA indicated that the density of astrocyte progenitors (Figure 8A) decreased with age (*F*_3,74_ = 12.4, *p* < 0.0001) and was higher in OXYS rats (*F*_1,74_ = 15.5, *p* < 0.0002). By contrast, *post hoc* analysis revealed a peak of astrocyte progenitor density in OXYS rats at PND20.

We found that astrocyte density was affected only by age (*F*_3,73_ = 9.2, *p* < 0.0001; Figure 8B). We observed that by PND20, astrocyte density decreased in Wistar and OXYS rats (*p* < 0.0004 and *p* < 0.02, respectively) with the decrease being less pronounced in OXYS rats: this parameter was higher in OXYS rats than in Wistar rats at PND20 (*p* < 0.0002). Until PND45, astrocyte density increased only in Wistar rats (*p* < 0.0001). The decrease of astrocyte density in the DG at PND20 may be caused by astrocyte migration to other cortical areas, whereas the less pronounced decrease of the parameter in OXYS rats may reflect alteration of the migration. Indirect evidence of such an alteration may be the finding that the density of astrocytes in the frontal cortex of Wistar rats at PND20 was the same as that at 3 months of age, whereas in OXYS rats, astrocyte density did not reach the adult level at PND20, remaining lower than at 3 months of age (Stefanova, unpublished data).

Regarding the distribution of astrocytic cells between DG layers (Figure 8C), we demonstrated that the percentage of astrocyte progenitors was lower in the molecular layer and higher in the hilus of OXYS rats (*F*_1,56_ = 23.8, *p* < 0.0001 and *F*_1,56_ = 22.5, *p* < 0.0001, respectively). This imbalance of distribution may point to altered migration of astrocyte progenitors in the DG of OXYS rats. Analysis of the astrocyte distribution (Figure 8D) uncovered an age-related moderate increase in the percentage of astrocytes in the granule zone and its moderate decrease in the subgranule zone (*F*_3,56_ = 3.6, *p* < 0.02 and *F*_3,56_ = 4.4, *p* < 0.007, respectively). Photomicrograph of astrocyte progenitors and astrocytes in DG is presented in the Figure 8E.

Taken together, the results were suggestive of altered migration of astrocytic cells from the DG in OXYS rats during postnatal brain formation.

## Discussion

It is only in the recent years that researchers turned their attention to the role of early stages of life in the development of AD and to the fact that the early postnatal period (which is marked by completion of brain maturation) may be when the first risk factors of subsequent cognitive impairment and accelerated aging (the main risk factor of AD) may form (Hall et al., 2015). Here we tried to evaluate the possible contribution—of alterations in the brain parameters reflecting its maturity at birth and in the period of postnatal development—to the development of AD-like pathology in OXYS rats.

The third trimester of pregnancy is especially important for development of the nervous system because the brain growth spurt, a peak of gliogenesis, and establishment of the blood–brain barrier occur in that period (Semple et al., 2013). Thus, alterations during the third trimester of pregnancy such as preterm birth (including first degree preterm birth, which usually is not followed by cognitive deficits) and trophic insufficiency during gestation may be among the determinants of the brain development trajectory and of the risk of neurodegenerative disorders (Heinonen et al., 2015). Developmental processes taking place in the third trimester of pregnancy in humans correspond to those during PND0–PND10 in rats (Semple et al., 2013). In the present work, we showed that the duration of gestation is shorter in OXYS rats than in Wistar rats by the period corresponding to 1.5 weeks in humans. Babies born 1.5 weeks earlier than term are not considered premature (Spong, 2013); therefore, we cannot conclude that OXYS rats are born preterm. Besides, OXYS pups are characterized by lower body weight at birth, however, by PND10, this parameter does not differ from that of Wistar rats. Thus, we can conclude that despite the lower body weight of female adult rats, OXYS pups are born underweight. Previously, we have observed a delay of development of postnatal reflexes and signs of retardation of hippocampal maturation in OXYS rats (Rudnitskaya et al., 2019), which may be consequences of shorter gestational duration. In the present study, we demonstrated a decrease of locomotor activity (specifically forelimb lifting) in OXYS rats already at PND10. Despite a natural increase in the activity of the animals from PND10 to PND45, the differences in locomotion between OXYS and Wistar rats intensified, with the locomotor activity of OXYS rats being significantly lower than that of Wistar rats. Also we revealed signs of increased anxiety in OXYS rats at PND45. The observed behavioral abnormalities may reflect alterations of brain formation during the postnatal period.

Neurogenesis is the key mechanism of brain development, and in this study, we identified specific features of neurogenesis in the hippocampal DG of OXYS rats. The formation of the hippocampal DG in rats starts on embryonic day 16, however, the majority (up to 85%) of granule cells matures after birth (Schlessinger et al., 1975; Altman and Bayer, 1990). Indeed, in rats, the peaks of the brain growth spurt and gliogenesis are seen during PND7–PND10 (Bockhorst et al., 2008; Kriegstein and Alvarez-Buylla, 2009), corresponding to the third trimester of pregnancy in humans. In rodents, the brain reaches 90–95% of adult weight by PND20–PND21, which corresponds to 2–3-year-old children. Additionally, this period is characterized by a peak of synaptic density, which in rats reaches a plateau at adult levels during PND35–PND49 corresponding to 12–18 years of age in humans (Semple et al., 2013). Consequently, in the present work, we focused on the PND0–PND45 period of brain development in OXYS and Wistar rats (Table 3). First, we found that the density of proliferating cells, QNPs and ANPs, in the DG of Wistar and OXYS rats naturally decreases from PND10 to PND14. These results together with a decrease in neuroblast density observed earlier (Rudnitskaya et al., 2019) reflect a decrease in neurogenesis intensity until PND14 in Wistar and OXYS rats. These results are consistent with the literature data, according to which the peak of rodent postnatal neurogenesis is reached at PND7 (Erecinska et al., 2004). Meanwhile, neurogenesis in the DG of OXYS rats is characterized by several features distinguishing them from Wistar rats. Namely, OXYS rats at PND10 are characterized by decreased density of ANPs, possibly because the enhanced neurogenesis generates greater numbers of neuroblasts and immature neurons at PND10 (Rudnitskaya et al., 2019), thus indicating the retardation of the postnatal peak of neurogenesis in the DG. Negative correlation between duration of gestation and QNPs density at PND10 indirectly confirms delay of peak of neurogenesis in OXYS rats. It should be mentioned that alterations of neurogenesis in early ontogenesis have been observed in an animal model of the familial form of AD: APPSwe/PS1ΔE9 mice (Lazarov and Demars, 2012). The density of ANPs in the DG of OXYS rats catches up to the level of Wistar rats between PND14 and PND20 and becomes higher at PND45. The increased ANP density at PND20 and PND45 may reflect faster neurogenesis as a compensation of its delay in OXYS rats and can result in increased density of mature neurons in the DG of OXYS rats at PND45 as detected previously (*p* < 0.05; unpublished data). The increase of neuronal density may be considered a compensation of the degenerative changes in the hippocampus of OXYS rats. Indeed, previously, we have documented increased neuronal and synaptic density against the background of an intensified activity of the neurotrophic system in OXYS rats at 3–5 months of age, with a dramatic decrease in the number of neurons and synapses and in neurotrophic activity by 18 months of age (Stefanova et al., 2015a, b; Rudnitskaya et al., 2017).

**TABLE 3:** A summary of key developmental events in the DG across comparable periods in humans and rats.

	Humans	Rats	Wistar rats	OXYS rats
Neurogenesis	From 3rd month of gestation	From E20-E21 (peaks fall at E21 and PND7)	Peak at PND10	Peak at PND10; increase by PND20
Gliogenesis	From 5th month of gestation to 1st postnatal year	PND1-PND20 (peak falls at PND10)	Peak at PND10	Peaks at PND10 and PND20
Synaptogenesis	From 6th month of gestation to 15th postnatal year	PND14-PND21 (peak falls at PND21)	Peak at PND21	Only IPB increased by PND21
Formation of SPB	From 3rd month of gestation	From E14 (at PND0, it is visible as a separate structure)	At PND0, it is visible as a separate structure	At PND10, it is visible as a separate structure

*E, embryonic day. The data for humans and rats are from: Abraham et al., 2004; Erecinska et al., 2004; Rahimi and Claiborne, 2007; Semple et al., 2013.*

After maturation, axons of granule cells start forming the SPB and IPB (Radic et al., 2017). Fibers of the SPB form mossy synapses on apical dendrites of pyramidal neurons in stratum lucidum, whereas the more variable IPB passes to stratum oriens and forms synapses on basal dendrites. Several axons from the IPB cross the pyramidal cell layer and adjoin the SPB. It has been reported that axons of adult-formed granular cells pass predominantly, but not exclusively, through the IPB (Römer et al., 2011). IPB length is determined genetically and correlates positively with good spatial learning (Crusio and Schwegler, 2005; Crusio et al., 2007; Delprato and Crusio, 2017). Hippocampal activity is also among the factors affecting IPB length: indeed, this length is greater in mice housed in an enriched environment as well as in animals with induced seizures (Römer et al., 2011). The length of the IPB decreases with age because of axonal retraction after the formation of mossy synapses on basal dendrites of pyramidal neurons. Normally, the decrease takes place from PND15 to PND45 (Bagri et al., 2003; Liu et al., 2005). Structural changes of mossy synapses have been found in J20 AD-transgenic mice (Wilke et al., 2014). Alterations in the functional activity of mossy fibers are observed in humans with neurodegenerative disorders and are considered some of the earliest signs of AD (Kobayashi, 2009; Mueller et al., 2010; Yassa et al., 2010, 2011). In the present work, we observed specific features of mossy-fiber formation in OXYS rats; these features indicate an alteration of normal development of synaptic networks between granule cells and pyramidal neurons of the CA3 area. This alteration may be a consequence of insufficient maturation of granule cells and their axons as well as the results of disturbances in the molecular mechanisms of extension and pruning of the axons forming mossy fibers, thus indicating a delay of DG development.

Astrocytic support is crucial for normal neuronal development (Reemst et al., 2016). We noted a gradual decrease in the density of astrocytic progenitors from PND10 to PND45 in Wistar and OXYS rats with the exception of increased progenitors’ density in OXYS rats at PND20. The observed increase may be a consequence of increased astrocytic demand among proliferating ANPs. Moreover, the distribution of astrocytic progenitors in the DG of OXYS rats differs from that of Wistar rats. In Wistar rats, the majority of astrocytic progenitors are located in the molecular layer of the DG, whereas in OXYS rats, they are located in the hilus. Such a distribution may be indicative of altered maturation and migration of astrocytic progenitors in OXYS rats (Reemst et al., 2016). Regarding the density of astrocytes, we demonstrated a decrease of this parameter at PND20 in Wistar and OXYS rats, which may reflect migration of astrocytes to other cortical areas. Nevertheless, in OXYS rats, this decrease was less pronounced; this finding may point to altered astrocytic migration. Indeed, in Wistar rats at PND20, astrocytic density in the frontal cortex was the same as that at 3 months of age, whereas in OXYS rats at PND20, this parameter was lower than that at 3 months of age (*p* < 0.01; unpublished data).

To summarize the obtained data, we documented alterations of hippocampal development in OXYS rats at an early age; however, these alterations got reversed as the animals became young adults (PND45). Our results are consistent with the observations of Zhuravin et al. (2004), who demonstrated restoration of some functions altered by perinatal hypoxia in young adult animals. Nonetheless, altered neuronal maturation and synaptic formation that are caused by perinatal hypoxia exert delayed effects on brain function late in life. Indeed, transgenic APPSwe/PS1A246E mice undergoing perinatal hypoxia exhibit accelerated manifestation of AD pathology (Zhang et al., 2013).

Thus, the shorter duration of pregnancy in OXYS rats may contribute to the delay of neurogenesis and of the formation of bundles of mossy fibers in the hippocampus as well as altered astrocytic migration, which results in decreased locomotor activity and signs of increased anxiety. We suppose that the observed features of early hippocampal development may be regarded as one of risk factors of AD-like pathology in OXYS rats.

## Data Availability Statement

The datasets generated for this study are available on request to the corresponding author.

## Ethics Statement

All the experimental procedures were in compliance with the Directive 2010/63/EU of the European Parliament and of the Council of 22 September 2010. The protocol of the animal study was reviewed and approved by the Commission on Bioethics of the Institute of Cytology and Genetics, SB RAS, Novosibirsk, Russia. Every effort was made to minimize the number of animals used and their discomfort.

## Author Contributions

NK, NS, and MS: conception and design of the experiments. TK and AB: work with animals and behavioral testing. ER: immunohistochemical analysis. AT and TP: visualization of mossy fibers. All authors drafting the manuscript or revising it critically for important intellectual content and approved the final version of the manuscript and agreed to be accountable for all aspects of the work in ensuring that questions related to the accuracy or integrity of any part of the work are appropriately investigated and resolved. All persons designated as authors qualify for authorship, and all those who qualify for authorship are listed.

## Conflict of Interest

The authors declare that the research was conducted in the absence of any commercial or financial relationships that could be construed as a potential conflict of interest.

## Footnotes

1 http://rgd.mcw.edu/

## References

[B1] Abraham H. L. Pérez-García C. G. Meyer G. (2004). P73 and Reelin in Cajal-Retzius cells of the developing human hippocampal formation. *Cereb. Cortex* 14 484–495. 10.1093/cercor/bhh010 15054064

[B2] Altman J. Bayer S. A. (1990). Migration and distribution of two populations of hippocampal granule cell precursors during the perinatal and postnatal periods. *J. Comp. Neurol.* 301 365–381. 10.1002/cne.903010304 2262596

[B3] Alzheimer’s Disease International (2019). *World Alzheimer Report 2019 Attitudes to Dementia.* London: Alzheimer’s Disease International.

[B4] Axelrud L. K. Sato J. R. Santoro M. L. Talarico F. Pine D. S. Rohde L. A. (2019). Genetic risk for Alzheimer’s disease and functional brain connectivity in children and adolescents. *Neurobiol. Aging* 82 10–17. 10.1016/j.neurobiolaging.2019.06.011 31376729 PMC7658444

[B5] Bagri A. Cheng H. J. Yaron A. Pleasure S. J. Tessier-Lavigne M. (2003). Stereotyped pruning of long hippocampal axon branches triggered by retraction inducers of the semaphorin family. *Cell* 113 285–299. 10.1016/s0092-8674(03)00267-8 12732138

[B6] Bateman R. J. Xiong C. Benzinger T. L. Fagan A. M. Goate A. Fox N. C. (2012). Clinical and biomarker changes in dominantly inherited Alzheimer’s disease. *N. Engl. J. Med.* 367 795–804. 10.1056/NEJMoa1202753 22784036 PMC3474597

[B7] Bockhorst K. H. Narayana P. A. Liu R. Ahobila-Vijjula P. Ramu J. Kamel M. (2008). Early postnatal development of rat brain: *in vivo* diffusion tensor imaging. *J. Neurosci. Res.* 86 1520–1528. 10.1002/jnr.21607 18189320

[B8] Crous-Bou M. Minguillón C. Gramunt N. Molinuevo J. L. (2017). Alzheimer’s disease prevention: from risk factors to early intervention. *Alzheimers Res. Ther.* 9:71. 10.1186/s13195-017-0297-z 28899416 PMC5596480

[B9] Crusio W. E. Genthner-Grimm G. Schwegler H. (2007). A quantitative-genetic analysis of hippocampal variation in the mouse. *J. Neurogenet.* 21 197–208. 10.1080/01677060701715827 18161583

[B10] Crusio W. E. Schwegler H. (2005). Learning spatial orientation tasks in the radial-maze and structural variation in the hippocampus in inbred mice. *Behav. Brain. Funct.* 1:3. 10.1186/1744-9081-1-3 15916698 PMC1143776

[B11] Delprato A. Crusio W. E. (2017). Genetic dissection of variation in hippocampal intra- and infrapyramidal mossy fibers in the mouse. *Methods Mol. Biol.* 1488 419–430. 10.1007/978-1-4939-6427-7_19 27933536

[B12] Encinas J. M. Michurina T. V. Peunova N. Park J. H. Tordo J. Peterson D. A. (2011). Division-coupled astrocytic differentiation and age-related depletion of neural stem cells in the adult hippocampus. *Cell Stem Cell* 8 566–579. 10.1016/j.stem.2011.03.010 21549330 PMC3286186

[B13] Erecinska M. Cherian S. Silver I. A. (2004). Energy metabolism in mammalian brain during development. *Prog. Neurobiol.* 73 397–445. 10.1016/j.pneurobio.2004.06.003 15313334

[B14] Hall V. J. Lindblad M. M. Jakobsen J. E. Gunnarsson A. Schmidt M. Rasmussen M. A. (2015). Impaired APP activity and altered Tau splicing in embryonic stem cell-derived astrocytes obtained from an APPsw transgenic minipig. *Dis. Model. Mech.* 8 1265–1278. 10.1242/dmm.019489 26398935 PMC4610230

[B15] Heinonen K. Eriksson J. G. Lahti J. Kajantie E. Pesonen A. K. Tuovinen S. (2015). Late preterm birth and neurocognitive performance in late adulthood: a birth cohort study. *Pediatrics* 135 e818–e825. 10.1542/peds.2014-3556 25733746

[B16] Hersi M. Irvine B. Gupta P. Gomes J. Birkett N. Krewski D. (2017). Risk factors associated with the onset and progression of Alzheimer’s disease: a systematic review of the evidence. *Neurotoxicology* 61 143–187. 10.1016/j.neuro.2017.03.006 28363508

[B17] Kobayashi K. (2009). Targeting the hippocampal mossy fiber synapse for the treatment of psychiatric disorders. *Mol. Neurobiol.* 39 24–36. 10.1007/s12035-008-8049-5 19130314

[B18] Kolosova N. G. Akulov A. E. Stefanova N. A. Moshkin M. P. Savelov A. A. Koptyug I. V. (2011). Effect of malate on the development of rotenone-induced brain changes in Wistar and OXYS rats: an MRI study. *Dokl. Biol. Sci.* 437 72–75. 10.1134/S0012496611020049 21562948

[B19] Kovács ÁM. Tauzin T. Téglás E. Gergely G. Csibra G. (2014). Pointing as epistemic request: 12-month-olds point to receive new information. *Infancy* 19 543–557. 10.1111/infa.12060 26568703 PMC4641318

[B20] Kriegstein A. Alvarez-Buylla A. (2009). The glial nature of embryonic and adult neural stem cells. *Annu. Rev. Neurosci.* 32 149–184. 10.1146/annurev.neuro.051508.135600 19555289 PMC3086722

[B21] Lazarov O. Demars M. P. (2012). All in the family: how the APPs regulate neurogenesis. *Front. Neurosci.* 6:81. 10.3389/fnins.2012.00081 22675290 PMC3366480

[B22] Lesuis S. L. Hoeijmakers L. Korosi A. de Rooij S. R. Swaab D. F. Kessels H. W. (2018). Vulnerability and resilience to Alzheimer’s disease: early life conditions modulate neuropathology and determine cognitive reserve. *Alzheimers Res. Ther.* 10:95. 10.1186/s13195-018-0422-7 30227888 PMC6145191

[B23] Liu X. B. Low L. K. Jones E. G. Cheng H. J. (2005). Stereotyped axon pruning via plexin signaling is associated with synaptic complex elimination in the hippocampus. *J. Neurosci.* 25 9124–9134. 10.1523/JNEUROSCI.2648-05.2005 16207871 PMC6725758

[B24] Morris J. K. Honea R. A. Vidoni E. D. Swerdlow R. H. Burns J. M. (2014). Is Alzheimer’s disease a systemic disease? *Biochim. Biophys. Acta* 1842 1340–1349. 10.1016/j.bbadis.2014.04.012 24747741 PMC4126236

[B25] Mueller S. G. Schuff N. Yaffe K. Madison C. Miller B. Weiner M. W. (2010). Hippocampal atrophy patterns in mild cognitive impairment and Alzheimer’s disease. *Hum. Brain Mapp.* 31 1339–1347. 10.1002/hbm.20934 20839293 PMC2943433

[B26] Nalivaeva N. N. Turner A. J. Zhuravin I. A. (2018). Role of prenatal hypoxia in brain development, cognitive functions, and neurodegeneration. *Front. Neurosci.* 12:825. 10.3389/fnins.2018.00825 30510498 PMC6254649

[B27] Radic T. Frieß L. Vijikumar A. Jungenitz T. Deller T. Schwarzacher S. W. (2017). Differential postnatal expression of neuronal maturation markers in the dentate gyrus of mice and rats. *Front. Neuroanat.* 11:104. 10.3389/fnana.2017.00104 29184486 PMC5694555

[B28] Rahimi O. Claiborne B. J. (2007). Morphological development and maturation of granule neuron dendrites in the rat dentate gyrus. *Prog. Brain. Res.* 163 167–181. 10.1016/S0079-6123(07)63010-6 17765718

[B29] Reemst K. Noctor S. C. Lucassen P. J. Hol E. M. (2016). The indispensable roles of microglia and astrocytes during brain development. *Front. Hum. Neurosci.* 10:566. 10.3389/fnhum.2016.00566 27877121 PMC5099170

[B30] Rodgers R. J. Dalvi A. (1997). Anxiety, defence and the elevated plus-maze. *Neurosci. Biobehav. Rev.* 21 801–810. 10.1016/s0149-7634(96)00058-9 9415905

[B31] Römer B. Krebs J. Overall R. W. Fabel K. Babu H. Overstreet-Wadiche L. (2011). Adult hippocampal neurogenesis and plasticity in the infrapyramidal bundle of the mossy fiber projection: I. Co-regulation by activity. *Front. Neurosci.* 5:107. 10.3389/fnins.2011.00107 21991243 PMC3180604

[B32] Rudnitskaya E. A. Kolosova N. G. Stefanova N. A. (2017). Impact of changes in neurotrophic supplementation on development of Alzheimer’s disease-like pathology in oxys rats. *Biochemistry* 82 318–329. 10.1134/S0006297917030105 28320273

[B33] Rudnitskaya E. A. Kozlova T. A. Burnyasheva A. O. Kolosova N. G. Stefanova N. A. (2019). Alterations of hippocampal neurogenesis during development of Alzheimer’s disease-like pathology in OXYS rats. *Exp. Gerontol.* 115 32–45. 10.1016/j.exger.2018.11.008 30415068

[B34] Schlessinger A. R. Cowan W. M. Gottlieb D. I. (1975). An autoradiographic study of the time of origin and the pattern of granule cell migration in the dentate gyrus of the rat. *J. Comp. Neurol.* 159 149–175. 10.1002/cne.901590202 1112911

[B35] Semple B. D. Blomgren K. Gimlin K. Ferriero D. M. Noble-Haeusslein L. J. (2013). Brain development in rodents and humans: identifying benchmarks of maturation and vulnerability to injury across species. *Prog. Neurobiol.* 106-107 1–16. 10.1016/j.pneurobio.2013.04.001 23583307 PMC3737272

[B36] Shimomura C. Ohta H. (1988). Behavioral abnormalities and seizure susceptibility in rat after neonatal anoxia. *Brain Dev.* 10 160–163. 10.1016/s0387-7604(88)80020-2 3407852

[B37] Sominsky L. De Luca S. Spencer S. J. (2018). Microglia: key players in neurodevelopment and neuronal plasticity. *Int. J. Biochem. Cell. Biol.* 94 56–60. 10.1016/j.biocel.2017.11.012 29197626

[B38] Spong C. Y. (2013). Defining “term” pregnancy: recommendations from the defining “term” pregnancy workgroup. *JAMA* 309 2445–2446. 10.1001/jama.2013.6235 23645117

[B39] Stefanova N. A. Ershov N. I. Maksimova K. Y. Muraleva N. A. Tyumentsev M. A. Kolosova N. G. (2019). The rat prefrontal-cortex transcriptome: effects of aging and sporadic Alzheimer’s disease-like pathology. *J. Gerontol. A Biol. Sci. Med. Sci.* 74 33–43. 10.1093/gerona/gly198 30265298

[B40] Stefanova N. A. Fursova A. Z. Kolosova N. G. (2010). Behavioral effects induced by mitochondria-targeted antioxidant SkQ1 in Wistar and senescence-accelerated OXYS rats. *J. Alzheimers Dis.* 21 479–491. 10.3233/JAD-2010-091675 20555140

[B41] Stefanova N. A. Kozhevnikova O. S. Vitovtov A. O. Maksimova K. Y. Logvinov S. V. Rudnitskaya E. A. (2014). Senescence-accelerated OXYS rats: a model of age-related cognitive decline with relevance to abnormalities in Alzheimer disease. *Cell Cycle* 13 898–909. 10.4161/cc.28255 24552807 PMC3984313

[B42] Stefanova N. A. Maksimova K. Y. Kiseleva E. Rudnitskaya E. A. Muraleva N. A. Kolosova N. G. (2015a). Melatonin attenuates impairments of structural hippocampal neuroplasticity in OXYS rats during active progression of Alzheimer’s disease-like pathology. *J. Pineal. Res.* 59 163–177. 10.1111/jpi.12248 25988948

[B43] Stefanova N. A. Maksimova K. Y. Rudnitskaya E. A. Muraleva N. A. Kolosova N. G. (2018). Association of cerebrovascular dysfunction with the development of Alzheimer’s disease-like pathology in OXYS rats. *BMC Genomics* 19:75. 10.1186/s12864-018-4480-9 29504901 PMC5836823

[B44] Stefanova N. A. Muraleva N. A. Korbolina E. E. Kiseleva E. Maksimova K. Y. Kolosova N. G. (2015b). Amyloid accumulation is a late event in sporadic Alzheimer’s disease-like pathology in nontransgenic rats. *Oncotarget* 6 1396–1413. 10.18632/oncotarget.2751 25595891 PMC4359302

[B45] Wilke S. A. Raam T. Antonios J. K. Bushong E. A. Koo E. H. Ellisman M. S. (2014). Specific disruption of hippocampal mossy fiber synapses in a mouse model of familial Alzheimer’s disease. *PLoS One* 9:e84349. 10.1371/journal.pone.0084349 24454724 PMC3890281

[B46] Yassa M. A. Lacy J. W. Stark S. M. Albert M. S. Gallagher M. Stark C. E. (2011). Pattern separation deficits associated with increased hippocampal CA3 and dentate gyrus activity in nondemented older adults. *Hippocampus* 21 968–979. 10.1002/hipo.20808 20865732 PMC3010452

[B47] Yassa M. A. Stark S. M. Bakker A. Albert M. S. Gallagher M. Stark C. E. (2010). High resolution structural and functional MRI of hippocampal CA3 and dentate gyrus in patients with amnestic mild cognitive impairment. *Neuroimage* 51 1242–1252. 10.1016/j.neuroimage.2010.03.040 20338246 PMC2909476

[B48] Zhang X. Li L. Zhang X. Xie W. Li L. Yang D. (2013). Prenatal hypoxia may aggravate the cognitive impairment and Alzheimer’s disease neuropathology in APPSwe/PS1A246E transgenic mice. *Neurobiol. Aging* 34 663–678. 10.1016/j.neurobiolaging.2012.06.012 22795785

[B49] Zhuravin I. A. Dubrovskaya N. M. Tumanova N. L. (2004). Postnatal physiological development of rats after acute prenatal hypoxia. *Neurosci. Behav. Physiol.* 34 809–816. 10.1023/b:neab.0000038132.08219.31 15587810

